# Therapeutic Potential of Mitophagy-Inducing Microflora Metabolite, Urolithin A for Alzheimer’s Disease

**DOI:** 10.3390/nu13113744

**Published:** 2021-10-23

**Authors:** Dona Pamoda W. Jayatunga, Eugene Hone, Harjot Khaira, Taciana Lunelli, Harjinder Singh, Gilles J. Guillemin, Binosha Fernando, Manohar L. Garg, Giuseppe Verdile, Ralph N. Martins

**Affiliations:** 1 Centre of Excellence for Alzheimer’s Disease Research & Care, School of Medical and Health Sciences, Edith Cowan University, 270 Joondalup Drive, Joondalup, WA 6027, Australia; p.jayatunga@ecu.edu.au (D.P.W.J.); e.hone@ecu.edu.au (E.H.); w.fernando@ecu.edu.au (B.F.); giuseppe.verdile@curtin.edu.au (G.V.); 2 Cooperative Research Centre for Mental Health, Carlton, VIC 3053, Australia; 3 Riddet Institute, Massey University, Private Bag 11222, Palmerston North 4442, New Zealand; h.khaira@massey.ac.nz (H.K.); t.lunelli@massey.ac.nz (T.L.); h.singh@massey.ac.nz (H.S.); manohar.garg@newcastle.edu.au (M.L.G.); 4 Department of Pharmacology, School of Medical Sciences, Faculty of Medicine, University of New South Wales, Sydney, NSW 2052, Australia; gilles.guillemin@mq.edu.au; 5 St. Vincent’s Centre for Applied Medical Research, Sydney, NSW 2011, Australia; 6 School of Biomedical Sciences and Pharmacy, Faculty of Health and Medicine, University of Newcastle, Callaghan, NSW 2308, Australia; 7 School of Pharmacy and Biomedical Sciences, Faculty of Health Sciences, Curtin Health Innovation Research Institute, Curtin University, Perth, WA 6102, Australia; 8 Australian Alzheimer’s Research Foundation, Ralph and Patricia Sarich Neuroscience Research Institute, 8 Verdun Street., Nedlands, WA 6009, Australia; 9 Department of Biomedical Sciences, Macquarie University, Sydney, NSW 2109, Australia

**Keywords:** Alzheimer’s disease, mitophagy, neuroprotection, nutraceuticals, pomegranate, urolithin A

## Abstract

Mitochondrial dysfunction including deficits of mitophagy is seen in aging and neurodegenerative disorders including Alzheimer’s disease (AD). Apart from traditionally targeting amyloid beta (Aβ), the main culprit in AD brains, other approaches include investigating impaired mitochondrial pathways for potential therapeutic benefits against AD. Thus, a future therapy for AD may focus on novel candidates that enhance optimal mitochondrial integrity and turnover. Bioactive food components, known as nutraceuticals, may serve as such agents to combat AD. Urolithin A is an intestinal microbe-derived metabolite of a class of polyphenols, ellagitannins (ETs). Urolithin A is known to exert many health benefits. Its antioxidant, anti-inflammatory, anti-atherogenic, anti-Aβ, and pro-mitophagy properties are increasingly recognized. However, the underlying mechanisms of urolithin A in inducing mitophagy is poorly understood. This review discusses the mitophagy deficits in AD and examines potential molecular mechanisms of its activation. Moreover, the current knowledge of urolithin A is discussed, focusing on its neuroprotective properties and its potential to induce mitophagy. Specifically, this review proposes potential mechanisms by which urolithin A may activate and promote mitophagy.

## 1. Introduction

Ageing is the time-dependent deterioration of cellular metabolic functions which has been characterized by several hallmarks. These include genomic instability, attrition in telomeres, loss of proteostasis, deregulated nutrient sensing, altered intercellular communication, cellular senescence, stem cell exhaustion, epigenetic alterations and mitochondrial dysfunction [1]. Ample evidence exists for a role for mitochondrial dysfunction in ageing [2,3,4,5]. In ageing and under stressful conditions, neurons exhibit an increase in oxidative phosphorylation (OXPHOS) by elevating glycolysis in astrocytes to meet the increased neuronal demand for lactate [6,7]. The increased OXPHOS on the other hand, results in an over- production of reactive oxygen species (ROS), namely superoxide (O_2_^−^) and hydrogen peroxide (H_2_O_2_). As per the mitochondrial free radical theory of ageing (MFRTA) proposed by Harman [8,9], aging is associated with oxidative stress exerted by high levels of ROS [1,10,11]. Mitochondrial nucleoids (mtDNA) located near the inner mitochondrial membrane (IMM), the sites of OXPHOS, are highly vulnerable to ROS-induced damage. Additionally, neurons, glial cells and microvessels in the brain transiently produce nitric oxide (NO) by activating various nitric oxide synthases (NOS) [12,13]. The reaction of NO with O_2_^−^ produces various reactive nitrogen species (RNS) including peroxynitrites (ONOO-). Oxidative stress occurs when the production of ROS overwhelms the antioxidant defences.

The protection afforded by the brain’s endogenous antioxidant defences are relatively low compared to other vital organs [14,15]. This makes the brain specifically susceptible to oxidative damage, since it also possesses high levels of fatty acids that readily oxidize oxidized transition metal species (Fe^3+^, Cu^2+^, Zn^2+^), and high mitochondrial activity to sustain its comparatively large energy demand [16]. In fact, accumulating mitochondrial bioenergetic abnormalities and mitochondrial damage in neurons may be central to many ROS-induced cellular pathological events in senescence and neurodegeneration, including the initiation and progression of Alzheimer’s disease (AD).

## 2. Alzheimer’s Disease

Alzheimer’s disease is the most common form of dementia that constitutes approximately 50–75% of clinically diagnosed dementia cases, worldwide [17]. This progressive neurodegenerative disorder is identified as the second major cause of death in Australia [18]. Furthermore, Asian [19] and Asia Pacific countries [17], were reported to have a tremendous increase in AD prevalence. It has been estimated that an 80-year-old has a 30% probability of getting the disease [20]. This higher prevalence rates and the universality of the disease may be dependent on various common conditions that impose a risk towards AD for which old age, female sex, and Apolipoprotein E allele status (*APOE ε4*) play key roles [21]. Lifestyle factors such as diet, head injury and smoking have also been shown to increase the risk of AD [22].

The key neuropathological features of AD are the accumulation of amyloid beta (Aβ) in extracellular senile plaques and intracellular neurofibrillary tangles made up of hyper-phosphorylated tau protein [23]. The final stage of AD brains also features cerebral atrophy [24]. Having no definite treatment to date, the major obstacle of AD diagnosis is that by the time clinical symptoms of cognitive deficits appear, the brain damage that has occurred is irreversible. Early diagnosis of the disease is critical, as there is a long prodromal/preclinical phase during which neuropathological changes are observed in the brain without clinical presentation [25,26]. It has been reported by Villemagne et al. (2013) that in AD patients, amyloid deposition in the brain occurs approximately two decades before the onset of clinical symptoms [27].

Oxidative stress accompanied by mitochondrial dysfunction is a characteristic feature in AD brains and is thought to be an early feature of the disease [28,29,30]. Amyloid beta directly enters mitochondria and disturbs mitochondrial function. Additionally, there is evidence that mitochondrial dysfunction arising from mitochondrion-derived ROS itself leads to aberrant Aβ production [31]. Therefore, coordinated mitochondrial biogenesis and elimination of dysfunctional mitochondria has the potential to play a pivotal role against AD.

## 3. Mitophagy Deficits in Alzheimer’s Disease

In living cells, selective degradation of defective mitochondria occurs in a pathway known as mitophagy. Mitophagy is a major mitochondrial quality control mechanism that ensures an efficient turnover to maintain a healthy population of mitochondria in cells. There are three major types of mitophagy: basal mitophagy, PINK1/Parkin-mediated mitophagy and mitochondrial vesicle-derived mitophagy [32]. Briefly, with the aid of various autophagy adaptor proteins, such as P62, Neighbor of BRCA1 gene 1 protein 1 (NBR1), Nuclear dot 10 protein 52 (NDP52/CALCOCO2), and Optineurin (OPTN), mitophagosomes are formed by damaged mitochondria, which are enclosed in double-membranes. These mitophagosomes mature and fuse with lysosomes, leading to enzymatic degradation of the contents and release of recycled materials into the cytosol. However, the efficiency of these mitophagy pathways declines with age [33]. Furthermore, impairment of mitophagy is increasingly recognized as a characteristic feature in AD brains [29].

It has been shown in mAPP mice that a lack of PINK1 promoted Aβ accumulation [34]. On the other hand, it has been reported that N-terminal truncated Tau induces aberrant Parkin recruitment, thus leading to excessive mitophagy and contributing to synaptic failure [35]. Previous studies have reported prominent autophagic accumulations of mitochondria in AD-affected brains [36,37,38,39]. This indicates that autophagic machinery is competent in AD neurons; however, the flux is impaired at the final stages of the turnover process, during the fusion of autophagosomes with lysosomes [40]. Moreover, in vitro studies have shown that hyper-phosphorylated Tau directly localizes and accumulates in mitochondria, leading to an increase in mitochondrial membrane potential and impaired PINK1/Parkin activation, resulting in deficits of mitophagy [41]. Parkin over-expression leads to an enhancement of the autophagic clearance of defective mitochondria [42].

Taken together, mitophagy deficits account for a significant part of the overall mitochondrial dysfunction as seen in AD. Therefore, inducing mitophagy confers neuroprotection against AD, as it ensures the maintenance of mitochondrial homeostasis [43]. Furthermore, an increasing number of review articles report that enhancing mitophagy holds promise as a novel therapeutic strategy for AD [44,45,46,47].

## 4. Enhancing Longevity: Molecular Pathways Leading to Mitophagy Activation

A build-up of high quantities of ROS in cells directly damage all biomolecules [48]. However, low levels of ROS give rise to an extended lifespan [49,50,51,52]. Whilst this initially seems counterintuitive, it is explained by a pathway termed mitohormesis [53]. Low levels of ROS activate Nuclear factor erythroid 2-related factor (Nrf2) in antioxidant response element (ARE)-mediated transcription of antioxidant proteins and phase I and II detoxification enzymes [54,55]. Moreover, the mitohormetic response promotes stress resistance, systemic antioxidant defence capabilities, and life span extension and is driven by a variety of cellular pathways and lifestyle interventions, which in turn activate the mitophagy process [55,56].

**Figure 1:**
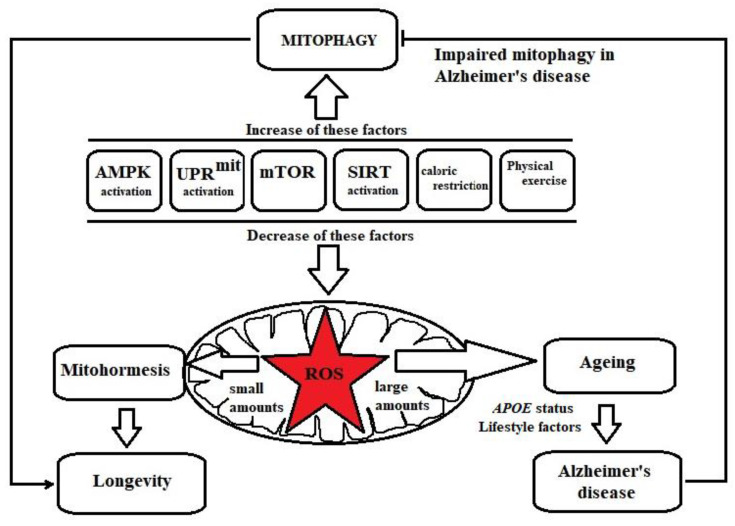
Factors regulating mitohormesis and mitophagy. Adenosine monophosphate (AMP)-dependent kinase (AMPK) signalling, mitochondrial unfolded protein response (UPRmit), Silent information regulator of transcription (Sirtuin) signalling, inhibition of mammalian target of rapamycin (mTOR), caloric restriction and physical exercise induce low levels of ROS in mitochondria. ROS, at low levels, activate stress resistance mechanisms (mitohormesis), resulting in longevity. However, increased amounts of ROS in mitochondria are responsible for ageing, which is a major risk factor for AD and is influenced by APOE status and a variety of lifestyle factors. AMPK signalling, UPRmit, Sirtuin signalling, mTOR inhibition, caloric restriction and physical exercise also induce the mitophagy process, which is defective in AD.

As shown in Figure 1, there are a variety of mitophagy modulatory pathways, namely, adenosine monophosphate (AMP)-dependent kinase (AMPK) signalling, mitochondrial unfolded protein response (UPRmit), Silent information regulator of transcription (Sirtuin) signalling and Mammalian targeting of rapamycin (mTOR). Moreover, caloric restriction and physical exercise are lifestyle interventions that contribute to the induction of mitophagy [57,58,59,60].

AMPK is an energy sensor in mitochondria that is regulated by the cellular AMP/ATP ratio. During energy deficits, the ratio of adenosine triphosphate (ATP)/AMP increases, stimulating AMPK activation [61]. The activation of AMPK requires specific phosphorylation events by upstream kinases such as the serine/threonine protein kinase known as liver kinase B1 (LKB1) [62]. AMPK inhibits anabolic pathways and increases catabolic processes to induce ATP generation. Acting as an initiator of mitochondrial biogenesis, AMPK induces mitochondrial metabolism to increase ATP levels via a mitohormetic response, which is based on low levels of ROS [63]. However, it is also reported that AMPK can also be activated by increased ROS levels [64]. The activated AMPK then phosphorylates mitochondrial fission factor (MFF) [65] and unc-51 like autophagy activating kinase 1 (ULK1) [66] to promote mitochondrial fission and subsequent mitophagy, respectively [67].

The UPRmit is a cellular response that occurs when there is an imbalance between nuclear DNA (nDNA)-encoded OXPHOS proteins and mtDNA-encoded OXPHOS proteins. This mitonuclear protein imbalance is characterized by accumulation of misfolded or unfolded proteins in the lumen of the endoplasmic reticulum (ER) [68]. The UPRmit induces mitochondrial protective genes including mitochondrial molecular chaperones and proteases and ROS defence antioxidants to re-establish protein homeostasis within the mitochondrial protein-folding environment [68,69]. Being a stress response mechanism, UPRmit is activated in neurodegenerative diseases including AD [70,71,72]. However, there is evidence that UPRmit drives mitophagy [58], despite the reports that mitophagy is impaired in AD [29]. Moreover, it has been shown in life span extension studies in C. elegans that UPRmit is induced by nicotinamide adenine dinucleotide (NAD^+^) boosters such as NAD^+^ precursors and poly ADP ribose polymerase (PARP) inhibitors by acting as co-factors in Sirtuin signalling [59,69].

The Sirtuins are a family of deacetylases (SIRT1-7) implicated in longevity [73,74]. Several studies indicate that they promote life span extension through the mitohormetic effect [75,76]. Redox reactivity of Sirtuins with NAD^+^ produce nicotinamide, which is methylated and is converted into 1-methylnicotinamide. This serves as a substrate for an aldehyde oxidase to produce hydrogen peroxide. The latter acts as a ROS signal to execute Sirtuin effects [77]. SIRT1 also suppresses the inducible nitric oxide synthase (iNOS) and thus, may decrease cellular ROS levels [78]. Primarily localized in the nucleus, SIRT1 translocation into mitochondria deacetylates the Peroxisome proliferation activator receptor gamma-co-activator 1α (PGC-1α), resulting in increased mitochondrial biogenesis [79]. Moreover, it has been demonstrated that SIRT3 promotes mitochondrial metabolic functions [80,81] and mitochondrial dynamics through activation of OPA1 to induce the fusion process [82]. In addition, SIRT2 deacetylation also leads to fork head box O (FOXO) transcriptional factor activation, resulting in the expression of various target genes including p27Kip1, manganese superoxide dismutase (MnSOD) and pro-apoptotic factor, Bcl-2 interacting mediator of cell death (Bim) [83]. FOXO activation by SIRT1 results in transcriptional activation of mitophagy [59].

The mTOR is another pathway involved in longevity [84,85,86]. It is a serine/threonine protein kinase that consists of two complexes, namely mTOR1 and mTOR2. The activity of mTOR1 is dependent on the accessory protein Regulatory-Associated Protein of mTOR (RAPTOR), which is thought to be a major cellular sensor of oxidative stress. Attenuation of mTOR signalling is associated with impaired glucose metabolism [87], resulting in mitohormesis and subsequent tissue-dependent modulation of mitobiogenesis [88]. There is evidence indicating that mTOR inhibition can lead to mitophagy [60,89].

Caloric restriction (CR) is reducing 10–50% ad libitum calorie intake without leading to malnutrition. It has been reported to be beneficial for healthy ageing and extending life span [90,91]. Additionally, agents that mimic the beneficial metabolic effects of CR are known as caloric restriction mimetics (CRMs). As evidenced in *Saccharomyces cerevisiae* and *C. elegans*, CR increases cellular OXPHOS levels, giving rise to elevated amounts of ROS and ultimately leading to longevity via hormetic ROS defence pathways [92,93,94]. On the one hand, as shown in rats, CR leads to the activation of SIRT1 and the suppression of mTOR [95]. On the other hand, CR has been shown to preserve the expression of SIRT3 and to increase the expression of AMPK and PGC-1α in skeletal muscle [96]. However, Lanza et al. (2012) [97] have shown that CR only preserves mitochondrial respiratory function rather than increasing mitochondrial abundance. In addition, a study conducted in rat cortical neurons has shown that CR-induced autophagy was stimulated by upregulated levels of neuropeptide Y (NPY) and ghrelin [98], a peripheral gut hormone that increases appetite. Additionally, Cui et al. (2013) [99] have confirmed that CR markedly increases the expression of autophagosome markers in kidneys of Fischer 344 rats. Apart from SIRT1/3 and AMPK activation and mTOR inhibition, which may lead to mitophagy, there is no direct evidence that CR regulates mitophagy. However, CR has been reported to exert neuroprotective effects [100].

An increasing number of studies has indicated the importance of exercise in longevity and healthy ageing [101,102,103]. One of the ways it is thought to exert its actions is through protection against agents that can cause cell death. It has been reported that exercise induces SIRT3 expression in cortical neurons, which deacetylates SOD2 and cyclophilin D in neuronal mitochondria, which provides protection against excitotoxicity [104]. Furthermore, during exercise, mitochondrial biogenesis is induced by increased PGC-1α levels [105,106]. However, a study by Philp et al. (2011) [107] indicates that upregulated PGC-1α levels are not due to the deacetylase activity of SIRT1, but are due to changes in the general control of amino-acid synthesis 5 (GCN5) acetyltransferase activity after exercise. Alternately, the high energy consumption during exercise disrupts the balance between AMP and ATP, leading to AMPK activation followed by induction of PGC-1α [108]. During exercise, PGC-1α promotes ROS generation via increased mitochondrial metabolism, leading to an elevated oxygen consumption in muscle fibres. This ROS generation is dependent on other exercise-induced changes in muscles, including increased CO2 tension, raised temperature and decrease in cellular pH [109]. However, PGC-1α has also been reported to induce ROS-detoxifying enzymes by Nrf2 activation [110]. In addition to the ROS-mediated adaptive responses, there is emerging evidence that exercise induces autophagy/mitophagy in skeletal muscle [111,112,113].

It has been reported that acute exercise induces mitophagy through AMPK-dependent activation of ULK1 in the skeletal muscle [66]. It has been reported that mitophagy occurs only at a later stage and not during or soon after high intensity endurance exercise [114]. The latter observation is consistent with the work of Ogborn et al. (2015) [115], who showed that the mitophagy proteins PINK1 and Parkin are not changed in response to exercise in the muscle from aged subjects immediately after a single bout of resistance training.

Despite the presence of the above-mentioned mitohormetic factors that promote stress resistance at low levels of ROS, the brain remains highly susceptible to oxidative stress induced by high levels of ROS. To counter this, the brain has several endogenous antioxidant defence mechanisms. These encompass the antioxidant enzymes: catalase, superoxide dismutase, superoxide reductase and glutathione peroxidase and antioxidants including glutathione (GSH), vitamin C and vitamin E. For supporting nearby neurons, astrocytes recycle vitamin C [116,117], and also maintain high concentrations of certain antioxidants by expressing Nrf2-dependent antioxidant genes. Moreover, neuronal inhibition of glycolysis stimulates the occurrence of the pentose phosphate pathway, which has significant implications for antioxidant defence due to the generation of the reduced form of nicotinamide adenine dinucleotide phosphate (NADPH), an essential cofactor for the generation of GSH [118]. However, these endogenous antioxidant defence mechanisms get overwhelmed, as ROS is continuously produced by vicious cycles due to Aβ-interactions with transition metal ions in AD brains.

Mitophagy induction has links to oxidative stress, as ROS induces the mitophagy process [119,120]. Therefore, in addition to exercise and caloric restriction, the lifestyle interventions so far known to induce mitophagy through pharmacological or nutraceutical interventions are extremely important.

## 5. Polyphenols with Potential to Induce Mitophagy

Given the lack of success of Aβ-targeted pharmaceutical approaches to date, the role of naturally occurring bioactive compounds as alternate treatment options for AD has gained considerable interest. Polyphenols in plant extracts are reported to play a role in delaying the onset of AD [121,122]. The most common types of polyphenols are phenolic acids, stilbenes, flavonoids and lignans. A major challenge for the use of these agents for therapy is thought to be their poor bioavailability after ingestion and inability to cross the BBB [123,124]. The most studied polyphenols that may be beneficial for AD include curcumin [125,126], resveratrol [127,128,129], quercetin [130,131], (-)-epigallactocatechin-3-gallate (EGCG) [132], and fisetin [133]. Many polyphenols exert favorable effects on mitochondria in terms of biogenesis and integrity [134]. However, the focus of this review is another group of nutraceutical compounds, urolithins, which have been shown to activate mitophagy [135].

## 6. Urolithins

Urolithins are 6H-dibenzopyran-6-one derivatives (or aglycons) produced by specific microbial transformations. The polyphenol ellagitannins (ETs) are the precursors of urolithins. ETs are hydrolysable tannins that occur in a wide variety of fruits such as pomegranates, strawberries, raspberries, blackberries and walnuts. Most importantly, pomegranate is a rich source of ETs and the most abundant ET in pomegranate is punicalagin. Its highest concentration is in the pomegranate peel, which is approximately 10.5 g/kg [136,137], while in the juice, it is between 1.5–1.9 g/L [138]. Hydrolysis of ETs to ellagic acid (EA) has been observed in the duodenum due to neutral pH conditions [139,140], whilst both ETs and EAs can undergo biotic changes in the lower gastro-intestinal tract [141].

### 6.1. Biological Effects of Urolithins

#### 6.1.1. Human Microbiota and Urolithin Metabotypes

Human microbiota is the entire collection of microorganisms living on the surface and the interior of the human body. Most of the microbiota live in the colon, where they exceed a density of 10^11^ cells per gram of bodyweight [142]. It is known that the human gut is colonized by hundreds of different species, where the species Firmicutes, Bacteroidetes and Actinobacteria are prominent [143]. However, the microbiome is highly dynamic and even within an individual, it is influenced by many factors such as age, diet, hormonal cycles, travel, illnesses, and usage of drugs, especially antibiotics. Most importantly, the gut microbiota composition plays a key role in the maintenance of human health [144].

Modulating the density of various bacterial communities in the gut is linked to both health improvement and deterioration. Beneficial bacteria, commonly known as probiotics (e.g., Bifidobacterium, Lactobacillus), function as a ‘barrier’ against pathogens. However, it is increasingly being observed that the composition of human gut microbiota is prone to changes in various diseases, including certain types of cancer [145] and also AD [146,147]. The role of microbiota in AD could be attributed to diet, which is being recognized to be a risk factor for AD [148,149]. Alterations in the gut microbiome have been found not only in rodent AD models, but recently also in human AD subjects. In one study, faeces of AD participants indicated decreased levels of Firmicutes and Bifidobacterium and increased levels of Bacteroidetes compared with those obtained from age- and gender-matched controls [146]. Interestingly, the different levels of these microbial genera have been observed to correlate with cerebrospinal fluid (CSF) biomarkers of AD [146].

There are specific dietary substrates that modulate the composition and metabolic function of the microbial communities. For instance, dietary phenolic and polyphenolic components of common foods readily contribute to gut bacteria modulation [150]. However, dietary fibre and prebiotics which remain undigested by human enzymes modulate the microbiota into specific gastrointestinal microbes, which generate short chain fatty acids (SCFAs) that are beneficial for human hosts [151]. Similarly, ETs in diet modify the gut microbiota including *Clostridium coccoides*, *Clostridium leptum* [152], or *Gordonibacter urolithinfaciens* [153], which transform the ETs into urolithins [154].

**Figure 2:**
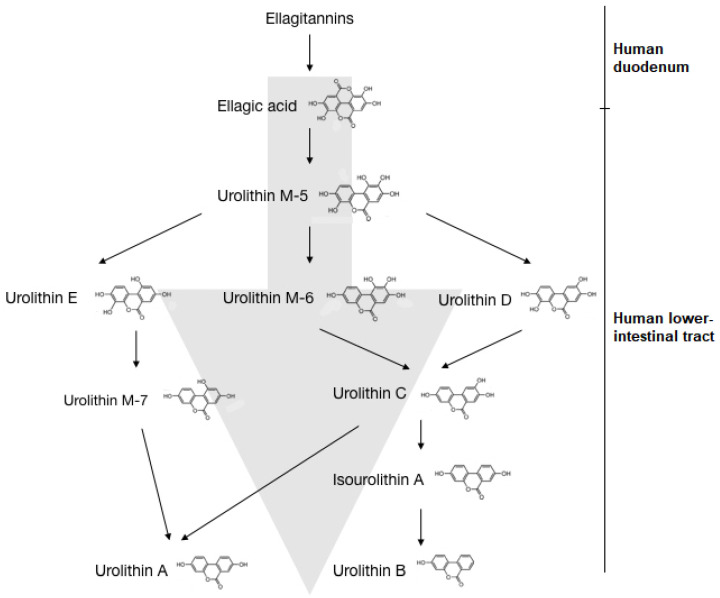
Bacterial transformation of ellagitannins into urolithin A and B in humans. The ETs in ingested food are converted to EA in the human duodenum. In the lower intestinal tract, EA is converted to urolithin M-5, which can be converted into the intermediates urolithin E, urolithin M-6 and urolithin D. Urolithin E converts to urolithin M-7, while urolithin M-6 and urolithin D give rise to urolithin C. Urolithin C converts to isourolithin A and urolithin B respectively. Alternatively, urolithin M-7 and urolithin C are converted to urolithin A.

As shown in Figure 2, ETs in ingested foods are converted into EA in the duodenum. EA then converts to urolithin D, retaining four phenolic hydroxyl groups. Subsequent metabolism continues along the gastrointestinal tract with the sequential removal of hydroxyl groups, leading to the production of urolithin C, urolithin A and urolithin B in the distal parts of the colon. Urolithin M-5, urolithin M-6, urolithin M-7, urolithin C and urolithin E are reported to be produced as side metabolites leading to urolithin A [152,155]. Urolithin A is shown to exert anti-ageing effects, increased mitochondrial activity and muscle function, potentially due to its mitophagy-inducing effects [135] and antioxidant effects [156]. Urolithin B is shown to act to reduce muscle atrophy [157], while lipid-lowering effects are exerted by urolithin C [158]. The rate of urolithin production is dependent on the type of microbiota and the ingested type of ET [159]. One study has shown that humans can be categorised into three metabolic phenotypes based on their ability to modulate microbiota, such that different urolithins are produced [160]. For example, after consuming an ET-rich diet, if an individual could excrete only urolithin A, he/she will be of metabotype A. Similarly, the ability to excrete urolithin B and/or isourolithin A defines metabotype B while metabotype O denotes the inability to produce urolithins [161].

Urolithin metabotypes have been reported to be potential cardiometabolic risk biomarkers [162]. The gut microbiota associated with obesity have been shown to have a link with a differential metabolism of EA and the urolithin-producing bacterial genus Gordonibacter [163]. However, further studies for urolithin metabotypes are required, as many of the basic facts including their induction, stability over time, diet preference and genetic and epigenetic influence remain largely unknown. Moreover, there are four new urolithin metabolites (urolithin M6R, M7R, CR, AR) which have been recently identified in human faeces and urine following intake of pomegranate extract, leading to further studies of unravelling gut microbial action [164].

#### 6.1.2. Bioavailability and Toxicity of Urolithins

Urolithins appear in the human circulation within a few hours of consumption of ET-containing foods, reaching maximum concentrations after 24–48 h and complete excretion in urine/faeces within 72 h. They are present in the plasma at very low concentrations ranging from 0.2–20 μM [165] and undergo further biotransformation, such as methylation, sulfation and glucuronidation, upon entering the enterohepatic circulation [166,167]. For oral and intravenous administration urolithin A, only glucuronidated and sulfonated phase II metabolites have been reported to be the prevalent metabolites from Absorption, Distribution, Metabolism and Excretion (ADME) studies [168]. It has been reported very recently that a phase II metabolism can also negatively affect certain pharmacological properties of urolithins [169].

Furthermore, liquid chromatography-electrospray ionization-tandem mass spectrometry (LC-ESI–MS/MS) has been recently utilised for the determination of urolithin C in rat plasma. In this study, glucuronyl and sulphate conjugates of urolithin C were the main metabolites detected in plasma [170]. In silico studies have predicted that urolithins can permeate the blood–brain barrier (BBB) [171]. Taken together with a report in which urolithin B has been detected in rat brains upon intravenous administration, this implies that urolithin A can enter the brain [172]. Furthermore, it has been reported very recently that urolithin A was detected in brains of a Parkinson’s disease rat model [173].

Urolithins are relatively non-toxic, as shown by studies in rats [172,173], as well as humans [174]. Furthermore, Urolithin A has been shown to be non-genotoxic by a battery of assays [168]. The lethal dose 50 (LD50) has been found to be greater than 5 g/kg body weight in rats for a pomegranate fruit extract standardized to consist of 30% punicalagins [175]. Another study has reported the effect of the pomegranate fruit extract in Wistar rats, at levels up to 600 mg/kg body weight for 90 days with no signs of toxicity [176]. In another toxicological study, rats were fed with chow diets with 6% punicalagin, where the daily intake of punicalagin ranged from 0.6 to 1.2 g. No evidence of toxicity was observed after feeding the rats with the punicalagin-enriched diet for 37 days [166]. Overall, their non-toxicity and relatively short half-life until excretion from the body are favorable characteristics for urolithins to be considered for use as potential therapeutic agents. However, a recent report hints at the possibility of urolithin A as a potential thyroid disruptor [177].

#### 6.1.3. Therapeutic Effects of Urolithins

Urolithins are rapidly being identified as possible therapeutic agents for many types of diseases, including cardiac dysfunction [178] and various cancers [179,180,181]. Urolithin C has been shown to exert triglyceride-lowering effects in both adipocytes and hepatocytes [158], as well as to induce apoptosis in PC12 cells through a mitochondria-mediated pathway [182]. Moreover, urolithin A, B, C and D have exerted different antiproliferative effects on human colon cancer cell lines [183].

Pomegranate has been shown to ameliorate inflammation in the gastrointestinal tract, owing to the presence of ETs, EA and the urolithins [184]. Furthermore, the gut health of the host can determine the effectiveness of pomegranate-derived compounds against inflammation. In a study of Fisher rats [185], urolithin A was found to be the most effective anti-inflammatory compound derived from pomegranate consumption. However, in rats with colon inflammation, the nonmetabolized ET-related fraction was more effective. The compounds in the peel of the fruit have also been shown to be effective. In a study conducted using a mouse model of obesity associated with hypercholesterolemia and inflammatory disorders, it was found that the symptoms were alleviated by pomegranate peel extract [186]. Moreover, urolithin A has been studied in a randomized, placebo-controlled, double blind clinical trial, which concluded that urolithin A is safe and bioavailable in humans and is effective against age-related muscle decline [187]. Another clinical trial has shown that UA at doses of 500 mg and 1,000 mg for 4 weeks modulated plasma acylcarnitines and skeletal muscle mitochondrial gene expression in elders [174].

### 6.2. Considerations for Urolithin A as a Therapeutic Agent for Alzheimer’s Disease

Due to their prominent roles in the disease process, Aβ accumulation, oxidative stress and inflammation are key therapeutic targets in AD. Evidence, primarily from in vitro studies, suggest a neuroprotective potential for urolithins via mechanisms that act upon these targets. The evidence presented in Table 1 indicates that not only can EA and punicalagin attenuate the neurodegenerative process, but it can also act upstream to reduce the accumulation of Aβ. There is evidence suggesting that these molecules inhibit BACE1 activity, leading to reduced Aβ production. A study based on activity-guided purification has revealed that EA and punicalagin have an inhibitory action on BACE1 [188]. Furthermore, other ETs, geraniin and corilagin from the plant *Geranium thunbergii* have also been shown to selectively inhibit BACE1 without modulating the activity of other common serine proteases [189].

No study has evaluated the potential of downstream ET metabolites, particularly urolithins, in the inhibition of Aβ production. Additionally, urolithins have been shown to inhibit Aβ fibrillization dose-dependently, as indicated by the Thioflavin T assay [171].

ETs and urolithins possess antioxidant properties due to the presence of phenolic hydroxyl groups in their chemical structure. Compared to urolithin A, EA, urolithin C and D have been reported to have a higher antioxidant potency, which was even higher than for vitamin C [200]. Moreover, the effect of punicalagin on oxidative stress has been investigated in rat intestinal epithelial cells (IEC-6) [201]. Its antioxidative properties are exerted by up-regulating the expression of HO-1 via a mechanism that involves PI3K/Akt activation and Nrf2 translocation [156].

As neuroinflammation is a key feature in AD [202,203], the compounds that show anti-inflammatory activity can be beneficial against AD. Pomegranate constituents have been reported to inhibit the enzymes cyclooxygenase (COX) and lipoxygenase (LOX) [184] that inhibit the formation of prostaglandins, which are the key modulators of inflammation. Furthermore, it has been demonstrated that pomegranate juice inhibits the p38-mitogen-activated protein kinase (p38 MAPK) pathway, subsequently decreasing the levels of transcription factor NF-κB and down-regulating the production of the proinflammatory mediators TNF-α, IL-1β and MCP1 [204]. These observations were confirmed by Olajide et al. (2014) [191] for punicalagin, including the inhibition of tumor necrosis factor receptor (TNFR)-associated factor 6 (TRAF-6) mediated neuroinflammation. Histone deacetylation also plays an important role in inflammation, as it is implicated in the deactivation of inflammation-related transcription factors NF-κB and AP-1. Inhibition of histone acetyltransferases (HATs) is another mechanism by which the urolithins exhibit anti-inflammatory properties [165].

ETs and urolithins can also promote neuroprotection via estrogen receptors, potentially through the activation of nuclear estrogen receptors (ERs) in the brain [205,206]. Due to their plant-based origin, ETs can be considered as phytoestrogens and the urolithin metabolites generated within the gut are referred to as enterophytoestrogens. Urolithins exhibit estrogenic or antiestrogenic properties, depending on the availability of endogenous estrogen (estradiol) and thus may be considered as selective estrogen receptor modulators (SERMs) [167,177,207]. Even among urolithins, urolithin A has been shown to exhibit a high affinity to ERs, specifically to ERα, compared to urolithin B [167]. Consistently, a more recent study has reported the expression of estrogen-regulated genes by urolithin A in an ERα-dependent pathway [208].

Hypercholesterolemia has a central role in AD [209,210]; therefore, modulation of cholesterol or enhancing anti-atherogenic effects may be beneficial against AD. Studies have shown that urolithins act as activation ligands for liver X receptors (LXRs) [190], which are involved in uptake, transport, efflux and excretion of cholesterol in a tissue-dependent manner [211]. EA also promotes cholesterol efflux in oxidized LDL-induced foam cells, inhibiting macrophage lipid uptake [212]. However, Mele et al. (2016) [190] showed that EA and urolithin C decrease the accumulation of cholesterol but do not promote cholesterol efflux in THP-1-derived macrophages.

As mitophagy deficits are key features of mitochondrial dysfunction in AD, safeguarding mitochondrial integrity by inducing mitophagy may potentially be an effective intervention for AD. In this respect, urolithins have been shown to restore mitochondrial homeostasis by improving mitochondrial function and biogenesis [213], and inducing mitophagy [135,214]. Furthermore, Webb et al. (2017) have reported that urolithin dose-dependently induced mitophagy in C2C12 myotubes [215]. However, research directed at an understanding of the underpinning mechanisms of urolithin-induced mitophagy is still in its infancy.

### 6.3. Insights on How Urolithin A May Trigger Mitophagy

**Figure 3:**
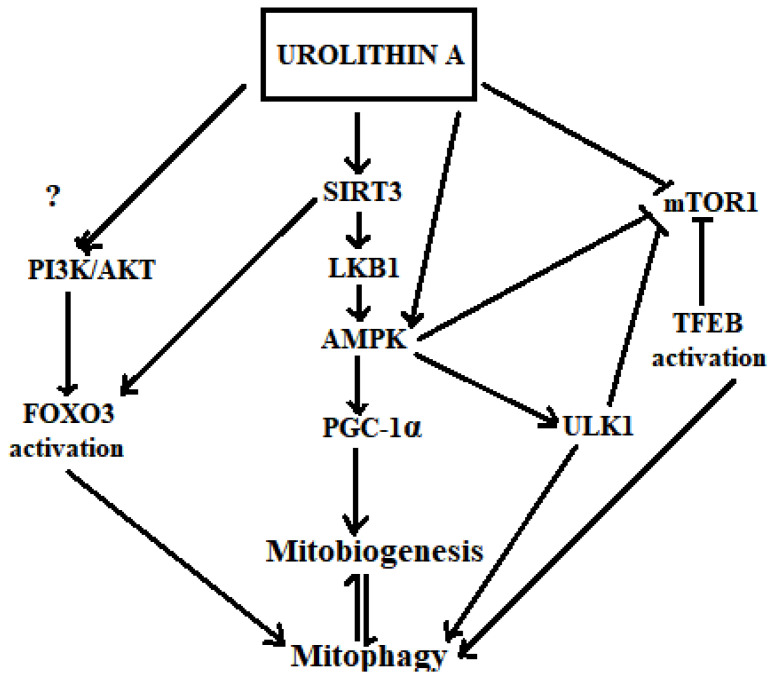
Hypothetical mechanisms of urolithin A in inducing mitophagy. Urolithin A activates SIRT1, SIRT3 [216] and AMPK. Activated AMPK increases PGC-1α levels [108] that directly increases mitochondrial biogenesis, which is in equilibrium with the mitophagy process. Activated AMPK also increases the activation of ULK1 [66]. Inhibition of mTOR1 is triggered by both urolithin A [217] and AMPK, which then activates ULK1 towards inducing mitophagy. The mTOR1 inhibition by Urolithin A may also induce the transcriptional activation of mitophagy via TFEBs. Urolithin A transcriptionally activates mitophagy via SIRT3-dependent FOXO activation [218,219].

As shown in Figure 3 and discussed further below, diverse evidence suggests that urolithins may modulate key signalling molecules in mitophagy and thereby regulate the activity of this pathway.

#### 6.3.1. Activation of SIRT1/3, AMPK, PGC1-α and Inhibition of mTOR1

Activation of SIRT, AMPK and PGC1-α and inhibition of mTOR tends to induce mitophagy and mitochondrial biogenesis to maintain a healthy mitochondrial population [220]. These key signalling pathways have been shown to be modulated by urolithins. SIRT deacetylates and activates LKB1, which is an upstream kinase that activates AMPK [221]. Zhao et al. (2016), have reported enhanced SIRT3 promoter activity in Caco-2 cells by urolithin A [216]. Urolithin A also increases ATP and NAD+ levels, leading to the activation of SIRT1 promoters, effecting the SIRT1-PGC-1α pathway. [222]. The SIRT1 activity modulates mitofusin 2 (Mfn2) expression and subsequent mitophagy [223]. It has also been shown that urolithin A increases Mfn2 in its pathway of inducing mitophagy [174]. Furthermore, activation of SIRT1 increases Parkin levels [224] and urolithin A (1000 mg) has been shown to transcriptionally increase Parkin and BECN1 levels after 28 days of treatment in humans [174].

Activation of AMPK is a broad checkpoint for many cell signalling pathways, including the induction of downstream mitophagy effectors such as ULK1 through the inhibition of mTOR1 [225,226]. Furthermore, urolithins’ ability to activate AMPK [227], and impair mTOR signalling [217], is reported. Therefore, these studies provide support that urolithins may induce AMPK-mediated activation of ULK1, which may then phosphorylate the LIR motif of FUNDC1 to promote mitophagy [174].

#### 6.3.2. Transcriptional Activation by TFEB and FOXO3

Transcription factor EB (TFEB) and FOXO3 are activated in response to low nutrient or energy status to upregulate the expression of autophagic and lysosomal genes [228,229,230]. In addition to their role in general autophagy, they have also been reported as key players in mitophagy [218].

The TFEB belongs to the helix-loop-helix-leucine-zipper (bHLH-Zip) class of microphthalmia-associated transcription factors (MiTFs) and regulates lysosomal biogenesis. The levels of TFEB in the nucleus reportedly corresponds to varying cellular demands for autophagosome-lysosome function [231]. TFEB binds to the coordinated lysosomal expression and regulation (CLEAR) motif. In the de-activated state, TFEB resides in the cytoplasm, recruited to the mTOR complex, which is localized in lysosomes to the cytoplasmic end [232]. Alternatively, mTOR1 inhibition promotes the translocation of TFEB to the nucleus and the downstream effect of mitophagy through transcriptional activity in a PINK1 and a Parkin-dependent manner [233,234]. There is no direct evidence to date that urolithins activate TFEB; however, pomegranate extract has been reported to activate TFEB independently of ERK1/2, mTORC1 and calcineurin [234]. This hints that urolithin A may also give rise to TFEB activation. This vision is further reinforced as TFEB activation also occurs independent of mTORC1 via Akt inhibition [235], and for the reports showing that urolithin A attenuates Akt signalling [165,199,236,237]. However, one exception is a report that urolithin A alleviates myocardial ischemia/reperfusion injury through activating the PI3K/Akt pathway [238].

On the other hand, SIRT3 deacetylates and activates FOXO3 to promote its nuclear translocation for transcriptional activation of PINK [239,240]. Furthermore, the activity of FOXO3 during the process of mitophagy has been evaluated in SH-SY5Y cells [219]. In this study, the levels of the mitophagy marker proteins Beclin 1, PINK and Parkin were shown to be increased, following the induction of mitophagy by MnCl2 and this was accompanied by FOXO3 nuclear retention. A more recent study carried out in SH-SY5Y and induced pluripotent stem cell (iPSC)-derived neurons, has also shown that Akt signalling increases mitophagy via regulating PINK 1 levels [241]. Additionally, FOXO3 is activated by the Phosphoinositide-3-kinase/ Protein kinase B (PI3K/Akt) pathway depending on the mTORC2 activation [242]. Therefore, due to controversy on the effect of urolithin A on the PI3K/Akt pathway, whether FOXO3 might be activated by urolithin A as a mode of transcriptional activation of mitophagy needs further evaluation.

## 7. Conclusions

The neurodegenerative processes that lead to AD remain to be fully understood and although the accumulation of Aβ and hyper-phosphorylated Tau protein have a key role, investigating the relevant cellular pathways that are impaired in the early stages of the disease process is required. Mitochondrial dysfunction is an early feature of the disease and although it is yet to be established as a cause or consequence of accumulating pathology, it is increasingly being recognized as a key contributor to the disease process. Avenues to attenuate or restore mitochondrial function could offer potential targets; however, an alternative is to promote removal of damaged/dysfunctional organelles. In this regard, enhancing the process of mitophagy is considered to be beneficial.

In addition to a greater understanding of disease mechanisms, it is increasingly apparent that any intervention for AD needs to be initiated as early as possible before the onset of irreversible neurodegeneration is to be effective. Due to their relative safety and efficacy compared to many other drugs in development, nutraceuticals are more attractive alternatives for the prevention and treatment of AD. Urolithins are particularly promising, as they represent microbial metabolites of ingested polyphenols and initial studies suggest that they may have a multifaceted therapeutic value for AD by their actions to reduce BACE1 activity, Aβ fibrillation, ROS damage, inflammation, and atherogenesis and most importantly, their ability to restore/induce mitophagy, which is impaired in AD. Furthermore, urolithin metabotypes warrant investigation as their impact on the microbiome may be an additional contribution to reducing AD risk.

## Figures and Tables

**Table 1:** Pomegranate/ellagitannins/urolithins-based studies in relation to neuroprotection.

Compound	*In Vitro* Model	*In Vivo* Model	Dose/ Duration	Neuroprotective Roles	Reference
Ellagic acid (EA), Urolithins A, B, C and D	Human umbilical vein endothelial cells (HUVECs)	-	10 µM	Anti-atherogenic effects	Mele et al., 2016 [190]
Punicalagin	Primary mixed glial cells, organotypic hippocampal slice cultures	-	5–40 µM	Anti-inflammatory effects	Olajide et al., 2014 [191]
Urolithin A/B	-	*C. elegans*	10 µg/mL	Anti-Aβ fibrillation effects	Yuan et al., 2016 [171]
Pomegranate (freeze dried)	SK-N-SH cells	-	50–200 ppm	Anti-inflammatory effects Inhibition of BACE1 and Aβ	Velagapudi et al., 2016 [192]
Pomegranate- (pulp hydroalcoholic extract juice, pulp aqueous extract)	PC12	-	800 μg/mL 6 h/12 h	Antioxidant activity Anti-apoptogenic activity	Forouzanfar et al., 2013 [193]
Pomegranate peel extract	-	Male C57Bl/6 mice	800 mg/kg/day for 35 days	Reduction of Aβ plaque density, lipid peroxidation Anti-inflammatory effects Increase in the expression of neurotrophin BDNF	Morzelle et al., 2016 [194]
Pomegranate extract	PC12	mice	800 mg/kg/day	Antioxidant effects Inhibition of Aβ-induced learning and memory deficiency	Choi et al., 2015 [195]
Pomegranate (freeze dried)	-	Transgenic mice APPsw/Tg2576	4% fruit diet	Antioxidant activity	Subash et al., 2014 [196]
Urolithin A/B	MCF7	-	40 µM	Estrogenic and anti-estrogenic activity	Larrosa et al., 2006 [140]
Urolithin A/B	SK-N-MC	-	10 μM	Antiglycative activity	Verzelloni et al., 2011 [197]
Urolithins A/B	BV2 murine microglia and SH-SY5Y non-contact, co-culture model	-	10 μM	Anti-neuroinflammatory activity	DaSilva et al., 2017 [198]
Urolithins A/B	BV2 murine microglia	-	3–30 μM	Anti-neuroinflammatory activity	Xu et al., 2018 [199]
